# Lefamulin harbors promising anti-tuberculosis activity against multidrug-resistant *Mycobacterium tuberculosis* isolates

**DOI:** 10.1128/spectrum.02250-25

**Published:** 2025-10-08

**Authors:** Jing Wu, Yuanfei Ji, Weihe Zhang, Siyi Chen, Yao Dong, Xia Yu

**Affiliations:** 1 National Clinical Laboratory on Tuberculosis, Beijing Key laboratory for Drug-resistant Tuberculosis Research, Beijing Chest Hospital, Capital Medical University, Beijing Tuberculosis and Thoracic Tumor Research Institute 12517, Beijing, China; Seton Hall University, South Orange, New Jersey, USA

**Keywords:** *Mycobacterium tuberculosis*, lefamulin, antimicrobial activity, intracellular activity

## Abstract

Multidrug-resistant tuberculosis (MDR-TB) is often associated with poor clinical outcomes. This study evaluated the *in vitro* activity of lefamulin (LEF) and intracellular activities against *Mycobacterium tuberculosis*. In this study, we evaluated the potential of LEF as a new drug candidate for treating *M. tuberculosis* infections, including MDR-TB. The antimicrobial susceptibility testing was performed to determine the minimum inhibitory concentrations (MICs) of LEF against 132 clinical isolates of *M. tuberculosis*. The intracellular activity of LEF and its interaction with other anti-tuberculosis drugs were also evaluated using *M. tuberculosis* H37Rv. From the 132 *M*. *tuberculosis* clinical isolates*,* the MIC_50_ and MIC_90_ were 0.5 µg/mL and 1 µg/mL, respectively. The tentative epidemiological cut-off (ECOFF) against LEF was defined at 1 µg/mL. After 5 days of incubation, LEF at 2 µg/mL inhibited 89.88% ± 1.73% of intracellular bacterial growth, which was comparable with the inhibitory rate of 94.29% ± 1.32% achieved by INH at 2 µg/mL. In addition, a synergy between LEF and bedaquiline (BDQ) was observed with a fractional inhibitory concentration index = 0.5. Furthermore, LEF showed no correlation with resistance to 10 anti-tuberculosis drugs. The minimum bactericidal concentration/MIC of LEF values suggested that it is a bacteriostatic drug against *M. tuberculosis*, and the bactericidal activity is mainly characterized by a concentration-dependent pattern. LEF has potent inhibitory activities against *M. tuberculosis in vitro* as well as in macrophages. Furthermore, the synergistic effect with BDQ also favors LEF as a promising drug candidate for tuberculosis treatment, especially for MDR-TB.

**IMPORTANCE:**

Lefamulin (LEF), the first systemic pleuromutilin antibiotic approved for human use, exhibits broad-spectrum activity against Gram-positive bacteria. However, its *in vitro* activity against *Mycobacterium tuberculosis* (*Mtb*) remains unexplored. This study evaluated the potential of LEF for treating *Mtb* infections, including multidrug-resistant tuberculosis. Our findings demonstrate that LEF possesses potent bacteriostatic activity against *Mtb in vitro* and exhibits synergistic effects when combined with bedaquiline. These results suggest LEF as a promising therapeutic candidate for tuberculosis treatment.

## INTRODUCTION

Tuberculosis (TB) remains the world’s leading cause of death from a single infectious disease, and 10.8 million new cases were estimated in 2023 worldwide by the World Health Organization (WHO) (1). Globally in 2023, 0.4 million people were estimated to have developed multidrug-resistant tuberculosis (MDR-TB), defined as resistance to isoniazid (INH) and rifampin (RIF) simultaneously, which is a persistent threat. Although several new drugs (i.e., bedaquiline [BDQ], delamanid [DLM], and pretomanid) have been developed for the treatment of drug-resistant TB over the past 50 years (2–4), which highlights the difficulties in developing new anti-TB drugs. Unfortunately, resistance to these antibiotics developed faster than expected, potentially as a result of pre-existing resistance in naïve strains (5). As treatment options for MDR-TB are limited and rapid emergence of resistance to new drugs, repurposing of the current antibiotics is promoted as a cost- and time-effective mechanism for providing new medicines.

Lefamulin (LEF) (Xenlta), previously known as BC-3781, is the first pleuromutilin antibiotic to display a broad spectrum of *in vitro* activity against Gram-positive and Gram-negative aerobic and anaerobic bacteria, and atypical bacteria, including resistant strains such as methicillin-resistant *Staphylococcus aureus* (MRSA), MDR *Streptococcus pneumoniae,* and macrolide-resistant *Mycoplasma pneumonia* (6–9). As the first pleuromutilin used systemically in humans, LEF has a unique mechanism of action that inhibits protein synthesis, that is, the pleuromutilin core and C-14 side chain of LEF can bind to the A- and P-sites of the 23S rRNA peptidyl transferase center in the 50S bacterial ribosomal subunit, preventing the proper positioning of tRNA CCA and interfering with the peptidyl transfer (10). LEF was approved for the treatment of community-acquired bacterial pneumonia (CABP) by the United States Food and Drug Administration and the European Commission in 2019 and 2020, respectively (11). A recent study showed LEF had no activity against *Mycobacterium abscessus in vitro* with a 90% minimum inhibitory concentration (MIC_90_) >32 μg/mL (12). Considered the nightmare of antibiotics, *M. abscessus* is notoriously difficult to treat and exhibits natural resistance to many antibiotics. The activity of LEF against *Mycobacterium tuberculosis* (*Mtb*) needed further evaluation.

In the present study, we determined the MICs of reference strains and clinical isolates of *Mtb* collected in Beijing, China, to better understand the antimicrobial activity of LEF against *Mtb*, especially MDR-TB. Furthermore, time-killing kinetic and intracellular killing experiments using LEF were performed against *Mtb*, whereas the bactericidal activity in macrophages was analyzed. Our results indicated that the repurposing of the current antibiotics can supply a meaningful strategy to screen therapeutic candidates for *Mtb* infections.

## MATERIALS AND METHODS

### Bacterial strains and culture conditions

*Mtb* reference strain H37Rv (ATCC 27294) and clinical isolated strains, including sensitive strains and MDR strains of *Mtb,* were stored in the Bio-bank in Beijing Chest Hospital. To determine the trends of LEF resistance, we conducted experiments at two time points across 10 years (2015 and 2024). A total of 67 non-MDR strains and 65 MDR-TB strains were randomly obtained in both 2015 and 2024. All strains were grown in Middlebrook 7H9 broth (BD Difco, USA) supplemented with 0.05% Tween 80 and 10% oleic acid-albumin-dextrose-catalase (OADC) Growth Supplement (BD Difco, USA) or on solid Middlebrook 7H10 medium (BD Difco, USA) supplemented with 10% OADC Growth Supplement (BD Difco, USA) at 37°C. All the strains were stored at −80°C freezer and were sub-cultured on solid Löwenstein–Jensen (LJ) medium (Encode, Zhuhai, China) at 37°C.

### MIC testing

LEF was purchased from Med ChemExpress (USA), and other antibiotics, including RIF, INH, BDQ, linezolid (LZD), and clofazimine (CFZ), were obtained from Solarbio (China). The broth microdilution method was performed according to the guidelines from Clinical and Laboratory Standards Institute (CLSI) (13). Middlebrook 7H9 Broth was used for the MIC test of *Mtb* strains. The final concentrations of LEF ranged from 0.031 to 32 μg/mL, after being serially diluted twofold in a 96-well microtiter plate. Bacteria were scraped from LJ medium, homogenized in a grinding bottle, and then adjusted to a McFarland turbidity of 1 with sterile saline before being diluted 20-fold for inoculation. Then, 100 µL of the diluted inoculum was added to each well. The plates were incubated at 37°C for 7 days. Subsequently, 30 µL of resazurin (0.2 mg/mL) solution was added to each well, and the plates were incubated for another 24 hours at 37°C, and the color development was observed. The MIC was defined as the lowest concentration of the drug that inhibited the color change of the chromogenic agent from blue to pink. RIF was used as a positive control, and the acceptable MICs of *Mtb* H37Rv (ATCC 27294) to RIF were 0.0625 µg/mL or 0.125 µg/mL in different batches (14).

### Bactericidal or bacteriostatic activity of LEF *in vitro*

The bacterial-drug mixtures with concentrations of 1 × MIC, 2 × MIC, 4 × MIC, 8 × MIC, 16 × MIC, and 32 × MIC from the MIC test plates described in MIC testing were taken and 100 µL medium from each well was serially diluted (by 10-fold) and inoculated onto 7H10 agar culture plates (supplemented with 10% OADC). The colony-forming units (CFUs) were counted after 3–4 weeks of inverted and incubated on these culture plates at 37°C (15). Determining the CFUs with relevance to detect 99.9% bacilli killed or a reduction in 3 log10 viable bacilli, which was considered the minimum bactericidal concentration (MBC) (16).

### Live/dead bacterial viability assay

The bacterial suspension was collected and adjusted to McFarland turbidity standard 1, and then diluted 20-fold for inoculation. The bacteria were co-incubated with LEF at concentrations of 1/8 × MIC, 1/4 × MIC, 1 × MIC, 4 × MIC, 8 × MIC, and 64 × MIC, with the dimethyl sulfoxide (DMSO) group serving as the negative control (NC). After 7 days, the bacterial OD_600_ was adjusted to 0.6, followed by incubation with 0.5% Tween 80 at 37°C for 30 minutes and washed twice with PBS. The bacteria were stained with 10 µg/mL PI (Solarbio) for 15 minutes, washed twice with phosphate-buffered saline (PBS), and then stained with 5 µg/mL 4′, 6-diamidino-2-phenylindole (DAPI) (LABLEAD) for 5 minutes. 100 µL of the stained bacterial suspension was added to a black 96-well plate in triplicate. Fluorescence values were measured using a multi-functional microplate reader (Synergy H1), with excitation wavelengths of 600–630 nm for propidium iodide (PI) and 350–405 nm for DAPI. For each condition, the percentage of PI-stained bacterial cells relative to DAPI-stained cells was calculated to determine the bacterial mortality rate (%).

### Time-kill curves

We evaluated the killing kinetic efficacy of LEF using standard strains of *Mtb* according to a design protocol previously described in the literature (15). Bacteria were inoculated at 5 × 10^5^ CFU in shaker tubes (12 mL) containing LEF or INH or BDQ (1/8 × to 64 × MIC); the tubes were incubated for 7 days at 37°C with oscillation (60 rpm). Bacteria were counted on days 0, 3, 4, 6, and 8 by inoculating serial dilutions on 7H10 agar plates (containing 10% OADC), and CFUs were counted by incubating the tubes at 37°C for an additional 3–4 weeks (17). All assays were performed in three replicates.

### Intracellular killing and concentration-kill assay

Each well of a 24-well plate was inoculated with 500 µL of THP-1 cells at a final concentration of 1 × 10^6^ cells/mL. The cells were induced to differentiate into macrophages with PMA (200 ng/mL) for 48 hours. *M. tuberculosis* (ATCC 27294) -infected THP-1 cells were incubated in RPMI 1640 medium containing 10% fetal bovine serum at a multiplicity of infection (MOI) of 1:1. Cells were gently washed three times with pre-warmed 1 × PBS to remove extracellular bacteria after 4 hours of infection at 37°C under 5% CO_2_. LEF and INH were tested individually at concentrations of 1, 2, and 4 µg/mL. For combination treatment, LEF and BDQ were co-administered at 1 µg/mL each. LEF and BDQ were also tested individually at 2 µg/mL. All compounds were added to their respective wells, and DMSO medium was used as the NC. Cell lysis was performed with 0.01% Triton X-100 at 1, 3, and 5 days for the LEF and INH single-drug groups, and at 1 and 3 days for the LEF + BDQ combination and single-drug groups, followed by gradient dilution with 1 × PBS. To quantify the number of CFUs, cells were cultured on 7H10 agar medium (supplemented with 10% OADC). Statistically, changes in the number of CFUs were observed when treated with LEF and BDQ compared to the initial control group (18).

### Checkerboard assay for compound interactions

The synergistic activity of LEF in combination with first-line anti-TB drugs was evaluated by previous studies using the *Mtb* H37Rv checkerboard test (19). The activity of LEF in combination with RIF, INH, BDQ, LZD, CFZ, and DLM was tested. The combination drug plate was prepared by adding 50 µL of 7H9 medium (with 10% OADC) to each well of a 96-well microtiter plate, and 100 µL of paracrine drug at a concentration of 16 × MIC was added to row H and diluted doubly to column A. The LEF was diluted from 1/8 × MIC to 16 × MIC, and then 50 µL was added to column 10 to column 1 in order of highest to lowest concentration to complete the drug plate for the combination experiment; bacterial solutions were prepared as described in MIC testing, and 100 µL was added to 96-well plates. Each combination was tested biostatistically in triplicate. After incubation of 96-well plates at 37°C for 7 days, indicators are added to read bacterial growth inhibition based on color change (20). The lowest dilution of each drug combination that demonstrated bacterial inhibition was used for the fractional inhibitory concentration index (FICI) determination; FICI was determined as the sum of the fractional inhibitory concentrations (FIC) of the individual antibiotics, as described in the following equation:

FICI = sum of FIC values for each antibiotic when acting alone;

FIC = CIC/MIC.

CIC = Combined Inhibitory Concentration, which indicates the lowest concentration of the antibiotic that inhibits growth in the combination;

MIC = the lowest concentration of the antibiotic that inhibits growth when acting alone;

The combination of antibiotics was considered as follows: synergy, ≤0.5; additive, 0.5–1.0; indifferent, 1.0–4.0; or antagonistic, >4.0.

### Correlation of resistance of LEF with 10 anti-TB drugs

Spearman’s correlation analysis was performed using IBM SPSS Statistics (Version 27) to assess the co-resistance patterns among 11 antimicrobial agents. The *P*-values were subsequently corrected using RStudio (version 2024.12.0 + 467). Additionally, the absolute values of correlation coefficients (|*r*|) were categorized into five tiers according to conventional interpretation guidelines: (i) negligible correlation: 0 ≤ |*r*| < 0.2; (ii) weak correlation: 0.2 ≤ |*r*| < 0.4; (iii) moderate correlation: 0.4 ≤ |*r*| < 0.6; (iv) strong correlation: 0.6 ≤ *|r*| < 0.8; and (v) very strong correlation: 0.8 ≤ |*r*| ≤ 1.

## RESULTS

### MIC distribution

The MIC value for the *Mtb* reference strain H37Rv was 0.5 µg/mL. LEF exhibited the potent antibacterial activity against the tested *Mtb* strains, including MDR-TB isolates, and the MIC of LEF was less than 1 µg/mL for most of the isolates (93.9%, 124/132 strains). Notably, 108 of 132 clinical strains had a MIC ≤0.5 µg/mL. Totally, the MIC_50_ and MIC_90_ values were 0.5 µg/mL and 1 µg/mL, respectively (Table 1). Against MDR-TB isolates, the MIC_90_ of LEF (0.5 µg/mL) was lower than non-MDR isolates (1.0 µg/mL).

**TABLE 1:** Distribution of lefamulin MICs among *M. tuberculosis* strains^*a*^^,^^*b*^^,^^*c*^^,^^*d*^

		*M. tuberculosis* clinical isolates with MIC (μg/mL)										
Period of strain isolation	Classification	0.03125	0.0625	0.125	0.25	0.5	1	2	4	Total	MIC_50_	MIC_90_
2015	Non-MDR	0	2	3	2	12	6	3	0	28	1	2
	MDR	1	2	9	8	4	2	1	0	27	0.25	0.5
	Total	1	4	12	10	16	8	4	0	55	1	2
2024	Non-MDR	1	4	7	18	5	4	0	0	39	0.5	1
	MDR	0	1	10	8	11	4	2	2	38	1	2
	Total	1	5	17	26	16	8	2	2	77	0.5	1
Overall	Non-MDR	1	6	10	20	17	10	3	0	67	0.5	1
	MDR	1	3	19	16	15	6	3	2	65	0.25	0.5
	Total	2	9	29	36	32	16	6	2	132	0.5	1

^*a*^ MIC_50_, concentration required to inhibit the growth of 50% of the isolates tested.

^*b*^ MIC_90_, concentration required to inhibit the growth of 90% of the isolates tested.

^*c*^ Non-MDR is clinically susceptible strains of *Mycobacterium tuberculosis*.

^*d*^ MDR is clinical strains of multidrug-resistant tuberculosis (MDR-TB) that are resistant to first-line drugs (rifampicin and isoniazid).

According to the distribution of MICs against LEF, we proposed a tentative epidemiological cut-off (ECOFF) at 1 µg/mL to define a LEF-resistant isolate. For the 8 *Mtb* strains with MIC ≥ 2 µg/mL, sequencing the *23S rRNA* revealed no mutant. Furthermore, comparative analysis of MIC distributions between clinical *Mtb* isolates collected in 2015 and 2024 showed that *Mtb* strains isolated in 2015 had higher MIC_50_ and MIC_90_ values (1 µg/mL and 2 µg/mL, respectively) compared to strains isolated in 2024 (0.5 μg/mL and 1 µg/mL). This suggests that no significant acquired resistance to LEF has emerged after market approval.

### MBC determination and live/dead bacterial viability assay

**Fig 1:**
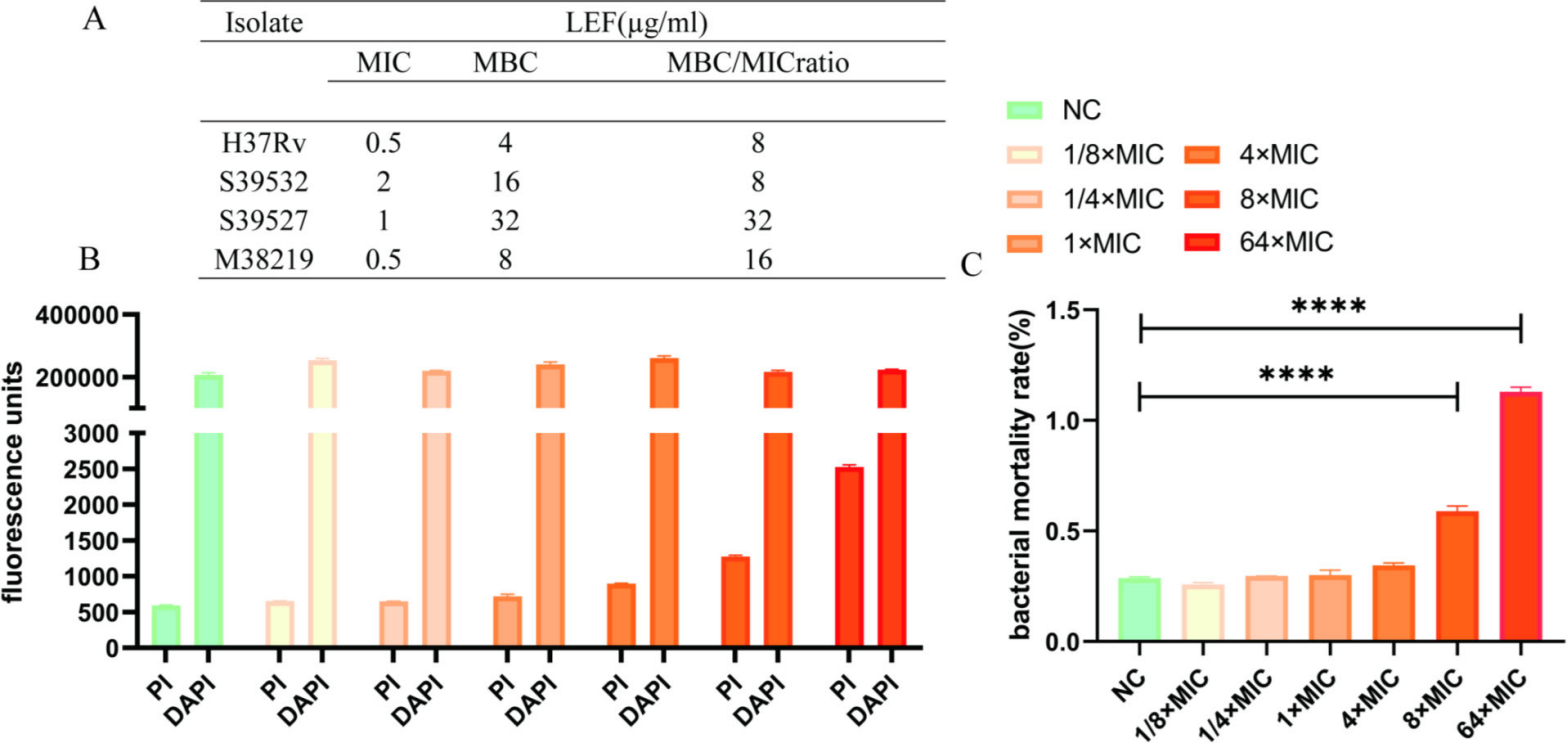
The bacteriostatic activity of LEF against *M. tuberculosis*. The MBC of LEF against *M. tuberculosis:* (**A**) the PI and DAPI fluorescence values (**B**) and bacterial mortality rate (**C**) of H37Rv after co-incubation with 1/8 × MIC, 1/4 × MIC, 1 × MIC, 4 × MIC, 8 × MIC, and 64 × MIC of LEF for 7 days are shown. All data are presented as the means ± SD (*n* = 3). ****P < 0.0001.

The MBC/MIC ratios of LEF against the *Mtb* reference strain H37Rv and three clinical isolates (including two pan-susceptible strains and one MDR-TB strain) ranged from 8 to 32 (Fig. 1A). Figure 1B shows a positive correlation between PI fluorescence intensity and LEF concentration. Notably, at concentrations ≥ 8 × MIC, bacterial mortality rates demonstrate statistically significant differences compared to the NC group (Fig. 1C). LEF exhibited bacteriostatic activity against *Mtb*, with MBC/MIC ratios > 4 and significant bacterial mortality at 8 × MIC.

### Time-kill assay

**Fig 2:**
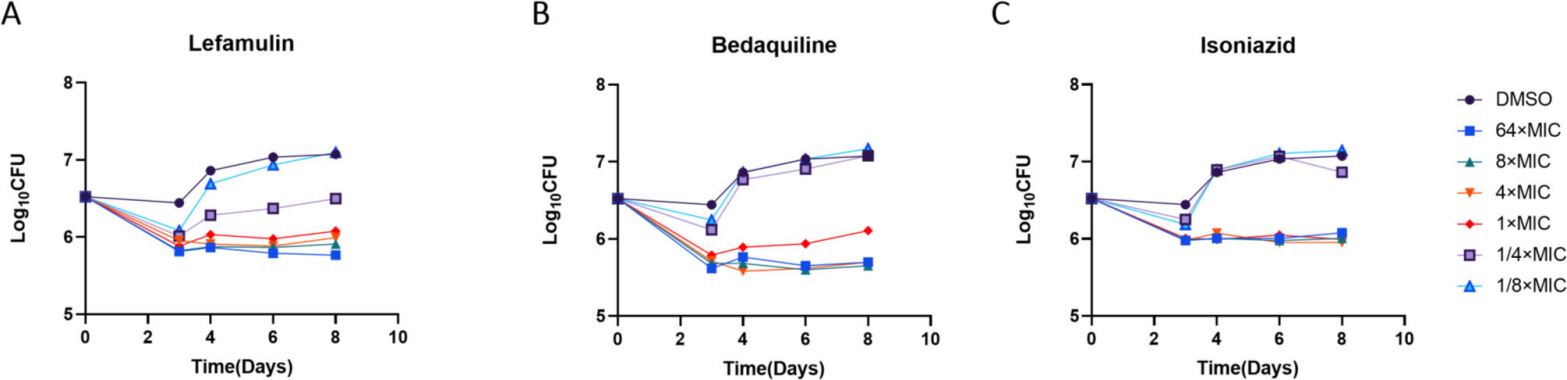
Time-kill curves of LEF (**A**), BDQ (**B**), and INH (**C**) against *M. tuberculosis* H37Rv. Antibiotic concentrations are presented as different symbols. The DMSO was used as a NC.

We further evaluated the antimicrobial activity of LEF using time-kill assays. In general, the bactericidal activity of LEF exhibits concentration-dependent characteristics. As shown in Fig. 2A, compared to the control group, LEF at concentrations ≥ 1 × MIC decreased bacterial counts by approximately 0.6 log_10_ CFU/mL by Day 3, which was slightly lower than the reduction observed with 1 × MIC of BDQ (Fig. 2B). However, at 4 × MIC, LEF achieved a reduction of nearly 0.5 log_10_ CFU/mL, comparable to the effect of 4 × MIC INH at Day 3 (Fig. 2C). During days 4–8, LEF at concentrations ≥ 1 × MIC effectively reduced bacterial load, with the maximal reduction observed on day 6. Overall, LEF ≥1 × MIC typically demonstrated better bactericidal activity compared to INH at ≥4 × MIC, which was slightly lower than that of BDQ at equivalent concentrations across all time points. These findings suggest that LEF, while showing somewhat less potent antimicrobial effects than BDQ, appears more effective than INH under the experimental conditions.

### Intracellular activity

**Fig 3:**
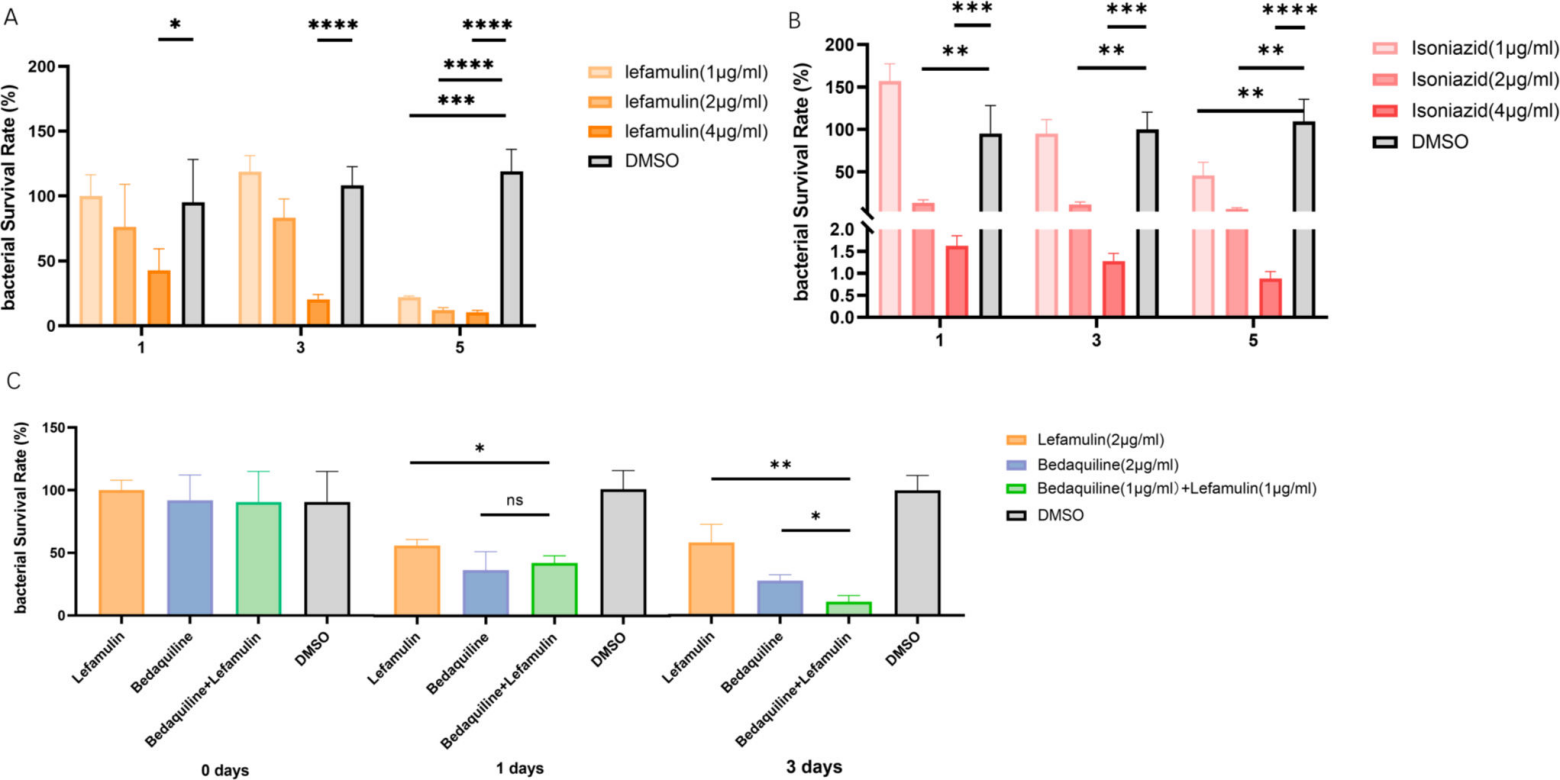
Intracellular bactericidal activities of LEF and BDQ using different concentrations against *M. tuberculosis* in THP-1. (**A**) LEF for *M. tuberculosis*, (**B**) INH for *M. tuberculosis*, and (**C**) intracellular survival of the combination of LEF and BDQ; All data are shown as the means ± SD (*n* = 3). *P < 0.05, **P < 0.01, ***P < 0.001, ****P < 0.0001.

Figure 3 shows the intracellular killing effect of LEF and/or combining with BDQ. Compared to DMSO control, the number of CFUs treated with LEF was reduced at an MOI = 1:1. After 5 days of incubation, the inhibitory rate of H37Rv by LEF at concentrations of 2 µg/mL and 4 µg/mL was 89.88% ± 1.73% and 91.11 ± 1.21%, respectively, which was comparable to that of INH at the corresponding concentration (94.29% ± 1.32%, and 99.19% ± 0.14%, respectively). Moreover, the inhibitory rate of LEF (81.48% ± 0.86%) was significantly higher than that of INH (58.22% ± 14.30%) at the concentration of 1 µg/mL (Fig. 3A and B). Synergistic effects in THP-1 macrophages became more evident by Day 3 but were not statistically significant on Day 1. After the treatment for 3 days, the drug combination group exhibited a significantly higher inhibitory rate compared to either BDQ or LEF alone, with inhibitory rates of 88.89% ± 4.81%, 72.22% ± 4.81%, and 41.67% ± 14.43%, respectively (Fig. 3C). This indicates that the antimicrobial effect of the combination of LEF and BDQ became substantially more pronounced over time.

### Combination studies

The synergy of LEF in combination with other clinical first-line anti-TB drugs was tested by checkerboard experiments. When combined with BDQ, LEF had a synergistic effect on H37Rv with FICI = 0.5. In addition, an additive effect with RIF, INH, LZD, CFZ, and DLM against *Mtb* H37Rv was found to have a FICI of 0.75 for INH and LZD, and a FICI of 0.625 for the rest of the drugs. No antagonistic interactions were found between LEF and the other tested compounds (Table 2).

**TABLE 2:** Combination of lefamulin with clinical first-line anti-tuberculosis drugs^*a*^

	MIC (μg/mL)			
Antibiotic combination	Antibiotics alone	Combinations	FICI	Outcome
LEF + RIF	0.5/0.00195	0.062/0.00097	0.625	Additive
LEF + INH	0.5/0.062	0.125/0.031	0.75	Additive
LEF + BDQ	0.5/0.062	0.125/0.016	0.5	Synergy
LEF + LZD	0.5/1	0.125/0.5	0.75	Additive
LEF + CFZ	0.5/0.5	0.062/0.25	0.625	Additive
LEF + DLM	0.5/1	0.062/0.5	0.625	Additive

^*a*^ MIC, minimal inhibitory concentration; FIC, fractional inhibitory concentration; FICI, fractional inhibitory concentration index; LEF, lefamulin; RIF, rifampicin; INH, isoniazid; BDQ, bedaquiline; LZD, linezolid; CFZ, clofazimine; DLM, delamanid; Synergy, FICI ≤ 0.5; Additive, 0.5 < FICI ≤ 1.0.

### Resistance correlation

**Fig 4:**
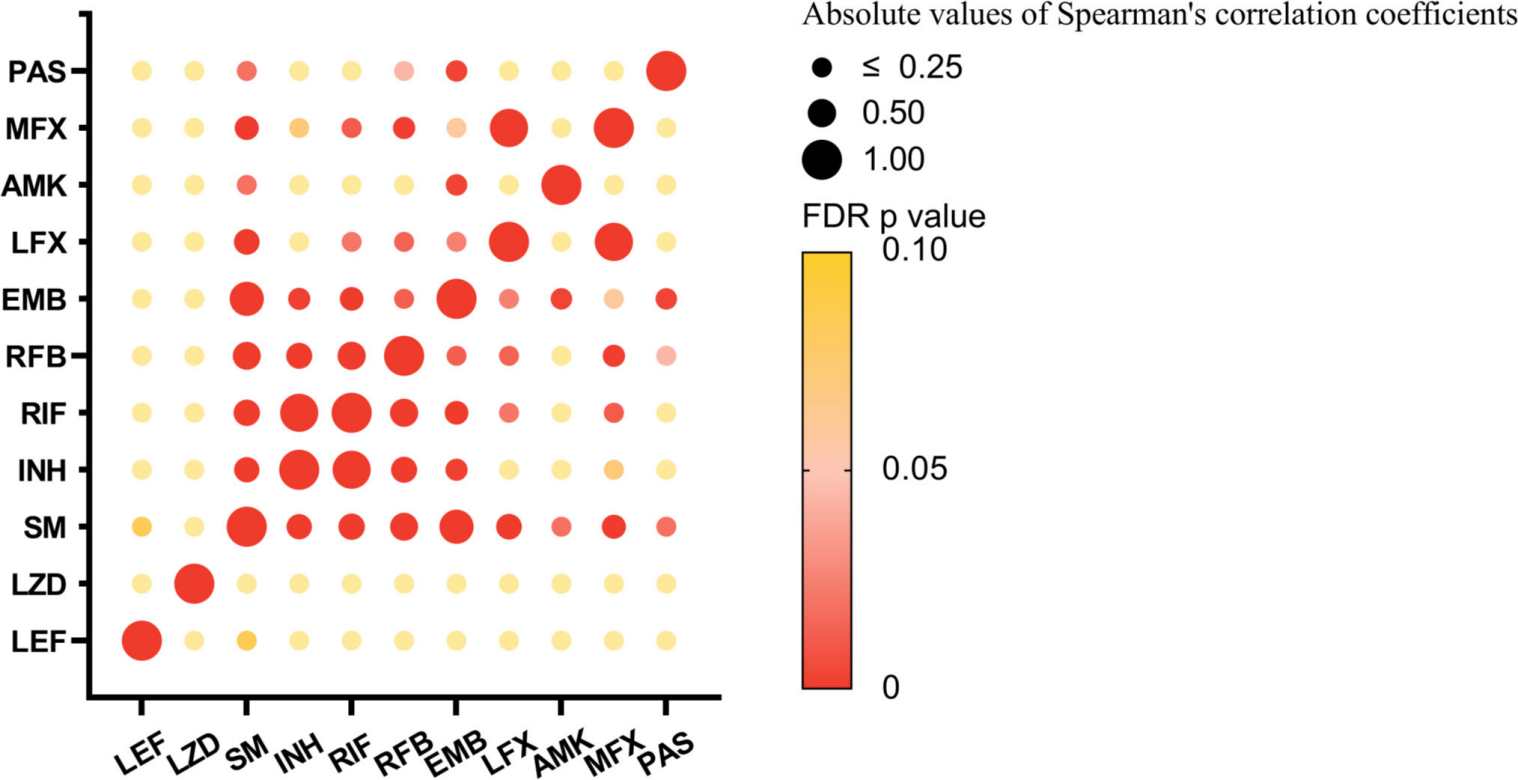
The correlation of resistance to 11 anti-TB drugs. Bubble plots showing the absolute values of Spearman’s correlation coefficients between the 11 drugs and the FDR-adjusted *P*-values, with the size of the dots indicating the magnitude of the absolute values of Spearman’s correlation coefficients and the color gradient indicating the FDR *P*-values (a significance threshold of FDR-adjusted *P*-values < 0.05 was applied).

The relevance of resistance to 11 anti-TB drugs was evaluated through bubble charts (Fig. 4). Except for the correlation between drugs in the same class, INH showed the strongest correlation with RIF (Spearman’s correlation ρ = 0.903). Moreover, SM is the drug most strongly associated with resistance to other agents, and it shows a high correlation with EMB resistance (Spearman’s correlation ρ = 0.726). Both EMB and RFB maintained relatively close correlations with other drugs, with EMB generally showing a weak correlation with other drugs. For LEF, none of the resistance correlations with 10 tested drugs were statistically significant after FDR correction for *P*, which means that LEF has greater potential for the treatment of MDR-TB.

## DISCUSSION

LEF showed potent activity against *S. pneumoniae* and *Staphylococcus,* evidenced by 100% inhibition at 0.25 µg/mL, and favorable MIC_50_/MIC_90_ (0.125/0.125 µg/mL) against *S. pneumoniae* (7), which aroused our interest in evaluating the potential of LEF as an anti-TB drug. In our study, LEF was determined to have antimicrobial activity against both the reference and clinical strains of *Mtb*. The MIC_50_ and MIC_90_ of LEF against *Mtb* strains were 0.5 µg/mL and 1 µg/mL, respectively. Importantly, LEF demonstrated good antimicrobial activities against both drug-susceptible *Mtb* and MDR-TB strains. Furthermore, LEF showed a synergy effect with the new anti-TB drug BDQ *in vitro* and THP-1, which might be important in regimen establishment for treating MDR-TB. Therefore, these outcomes suggest that LEF might be a promising candidate for the treatment of TB, including MDR-TB.

LEF demonstrated potent *in vitro* activity against a wide range of pathogens commonly associated with CABP, including *S. pneumoniae*, *Haemophilus influenzae*, *S. aureus* (including drug-resistant strains), and atypical bacteria (21, 22). For instance, 99.8% of *S. aureus* isolates and 99.3% of beta-hemolytic *Streptococcus* spp. were inhibited by LEF at ≤0.5 µg/mL, while 88.1% of *E. faecium* isolates showed inhibition at ≤1 µg/mL (23). The susceptible breakpoint for LEF was ≤0.5 µg/mL and ≤2 µg/mL for *S. pneumoniae* and *H. influenzae*, respectively (24, 25). Although generally not considered a pathogen for CABP, *Mtb* may occasionally coexist with CABP (26, 27). From the MICs distribution of LEF against 132 *Mtb* isolates, the tentative ECOFF was proposed as 1 µg/mL, which is comparable to the breakpoint of *H. influenzae* and *S. pneumoniae*. These findings suggest that LEF may also have therapeutic potential against TB.

Although LEF had not shown bactericidal activity *in vitro*, it manifested strong antibacterial activity in the intracellular bactericidal experiment in our study. As a pleuromutilin derivative, LEF has been shown to exhibit high intracellular concentrations in macrophages and demonstrates strong penetration capability (28). Consistent with previous studies, our study showed LEF (2 µg/mL) inhibited 89.88% ± 1.73% of intracellular bacterial growth at MOI = 1 after the treatment of 5 days, which was comparable with INH at 2 µg/mL. However, the actual antibacterial effects of drugs *in vivo* often differ from *in vitro* results due to complex factors, including variations in tissue blood flow, physiological barriers, diverse cell types, and pathological changes. In TB, the complex cellular composition of granulomas, hypoxia, and caseous necrosis poses challenges to drug diffusion and activity (29). Therefore, intracellular experiments alone cannot fully assess LEF’s antibacterial effects, and further studies are needed to evaluate this drug’s efficacy against TB in animal models.

LEF, which can be administered both orally and intravenously, presented *in vivo* activity against CABP. In adults, 5-day oral LEF showed a noninferior early clinical response at 96 hours compared to 7-day oral moxifloxacin (30). Previous studies provided support for LEF 150 mg intravenous (IV) q12h or 600 mg orally q12h for the treatment of patients with CABP and suggest that doses may not need to be taken under fasted conditions (31). Administration of the drug at therapeutic doses, the maximum plasma concentration (C_max_) of LEF in healthy adults following a single 150 mg IV infusion was 1.9–2.92 μg/mL, whereas under fasting conditions, a single 600 mg oral dose achieved a C_max_ of 1.2–1.5 μg/mL (32–34). In adults with cystic fibrosis (CF), the C_max_ values of LEF were 1.92 µg/mL with a 150 mg 1 hour IV infusion and 1.31 µg/mL with a single 600 mg oral dose under fasted conditions (35). Under any of the aforementioned administration conditions, LEF achieves a C_max_ surpassing its MIC_90_ (1 µg/mL) against *Mtb*, suggesting its promising therapeutic efficacy *in vivo*. In addition, the population pharmacokinetic (PPK) model also demonstrated excellent penetration of LEF, with a total-drug epithelial lining fluid (ELF) AUC_0-24_/free-drug plasma AUC_0–24_ ratio of approximately 5:1 following either IV or oral administration. Such penetration capacity markedly surpasses the AUC ratios reported for levofloxacin (1.59–2.69) and LZD (2.3–4.2) (36, 37). This indicated that LEF can rapidly penetrate target sites such as alveolar spaces. Taken together, the evidence conclusively demonstrates LEF’s therapeutic potential against TB.

LEF was found to exhibit no significant toxicity in normal human hepatocytes (L02 cells) compared to its effects on hepatocellular carcinoma cells (38). In addition, clinical trial data indicate that LEF is generally well tolerated. The most common adverse effects (AEs) of LEF for CABP were infusion-site reactions and diarrhea (10). Both clinical trials met efficacy endpoints for non-inferiority and provided evidence that LEF was generally well tolerated, with serious treatment-emergent adverse events occurring in <5% of patients (39). In CF patients, LEF exhibited similar AE patterns to those observed in healthy volunteers following both oral and IV administration. The treatment was considered safe in clinical use, with only mild to moderate AEs reported. No clinically significant abnormalities were found in physical examinations, vital signs monitoring, or electrocardiogram readings during the study period (35). Consistent with prior reports, across four PPK studies investigating different oral or IV administration routes, all AEs associated with LEF were mild to moderate in severity, primarily manifesting as gastrointestinal symptoms. No participants discontinued the studies due to AEs (34). These studies collectively demonstrate that LEF has an acceptable safety profile.

There are some limitations to our study. First, all the tested *Mtb* isolates were collected from a single hospital, Beijing Chest Hospital, which may be genetically and epidemiologically linked. More clinical isolates are required to evaluate *in vitro* LEF susceptibility with varying epidemiological backgrounds. Second, parts of *Mtb* strains exhibit higher MICs (≥ 2 µg/mL) without *23S rRNA* mutations, indicating potential alternative resistance mechanisms that warrant further study. Elevated LEF MICs (≥ 2 µg/mL) in some *Mtb* strains lacking *23S rRNA* mutations suggest alternative resistance mechanisms. Efflux pump activation through ABC transporters, MFS, and RND families may contribute, alongside dynamic remodeling of the mycolic acid-rich cell wall that reduces permeability (40). Thus, the specific resistance mechanisms of LEF against *Mtb* require further investigation. Third, our study evaluated the antibacterial effects of LEF against *Mtb* only at the *in vitro* and intracellular levels, and future studies involving animal models will be required to evaluate the pharmacokinetics and pharmacodynamics of LEF in TB treatment.

In conclusion, LEF exhibits strong inhibitory activity against *Mtb in vitro* and in macrophages. In addition, LEF is a promising drug candidate for TB treatment due to its synergistic effect with BDQ. Our data provided important insights into the potential clinical applications of LEF in treating TB infections. Further *in vivo* studies are essential to comprehensively evaluate therapeutic efficacy against TB and assess long-term safety.

## Data Availability

All data relevant to this study are available from the corresponding author upon reasonable request.
